# Genetic Characterization of *Vibrio cholerae O1* isolates from outbreaks between 2011 and 2015 in Tanzania

**DOI:** 10.1186/s12879-017-2252-9

**Published:** 2017-02-20

**Authors:** Yazid Kachwamba, A. A. Mohammed, H. Lukupulo, L. Urio, M. Majigo, F. Mosha, M. Matonya, R. Kishimba, J. Mghamba, J. Lusekelo, S. Nyanga, M. Almeida, S. Li, D. Domman, S.Y. Massele, O. C. Stine

**Affiliations:** 1 Muhimbili University of Health and Allied Sciences, Dar es Salaam, United Republic of Tanzania; 2 Tanzania Field Epidemiology and Laboratory Training Program, Dar es Salaam, United Republic of Tanzania; 3 National Health Laboratory, Quality Assurance and Training Centre, Dar es Salaam, United Republic of Tanzania; 4 Tanzania Ministry of Health, Community Development, Gender, Elderly and Children, Dar es Salaam, United Republic of Tanzania; 5 Center for Bioinformatics and Computational Biology, University of Maryland, College Park, Maryland USA; 6 Department of Epidemiology and Public Health, University of Maryland, Baltimore, Maryland USA; 7 Wellcome Trust Sanger Instititue, Hinxton, England

## Abstract

**Background:**

Cholera outbreaks have occurred in Tanzania since 1974. To date, the genetic epidemiology of these outbreaks has not been assessed.

**Methods:**

96 *Vibrio cholerae* O1 isolates from five regions were characterized, and their genetic relatedness assessed using multi-locus variable-number tandem-repeat analysis (MLVA) and whole genome sequencing (WGS).

**Results:**

Of the 48 MLVA genotypes observed, 3 were genetically unrelated to any others, while the remaining 45 genotypes separated into three MLVA clonal complexes (CCs) - each comprised of genotypes differing by a single allelic change. In Kigoma, two separate outbreaks, 4 months apart (January and May, 2015), were each caused by genetically distinct strains by MLVA and WGS. Remarkably, one MLVA CC contained isolates from both the May outbreak and ones from the 2011/2012 outbreak in Dar-es-Salaam. However, WGS revealed the isolates from the two outbreaks to be distinct clades. The outbreak that started in August 2015 in Dar-es-Salaam and spread to Morogoro, Singida and Mara was comprised of a single MLVA CC and WGS clade. Isolates from within an outbreak were closely related differing at fewer than 5 nucleotides. All isolates were part of the 3^rd^ wave of the 7^th^ pandemic and were found in four clades related to isolates from Kenya and Asia.

**Conclusions:**

We conclude that genetically related *V. cholerae* cluster in outbreaks, and distinct strains circulate simultaneously.

**Electronic supplementary material:**

The online version of this article (doi:10.1186/s12879-017-2252-9) contains supplementary material, which is available to authorized users.

## Background

*Vibrio cholerae* is the causative agent of cholera, a secretory diarrheal disease resulting in high mortality among humans, if untreated [1]; cholera continues to be a significant public health problem in countries with poor socio-economic conditions. The burden of this disease is estimated to be 3–5 million cases, with 100,000–120,000 deaths annually [2], and the number of cholera cases reported to the World Health Organization continues to rise. In 2014, 42 countries reported a total of 190,549 cholera cases and 2,231 deaths — an overall case fatality rate of 1.17% [2]. About 85% of the cases occur in Africa and southern Asia [2].

Since an initial report in 1974, cholera outbreaks have been reported regularly in Tanzania [3]. Outbreaks have been reported in various regions, including Dar es Salaam, Dodoma, Kigoma, Lindi, Mbeya, Morogoro, Mtwara, Pwani and Tanga; case fatality rates (CFR) have ranged from 1.3% to 11.7% [3]. Between 2011 and 2016 seven outbreaks have been reported from six regions, including Dar es Salaam, Kigoma, Morogoro, Singida, Tanga, and Mara.

It has been difficult to determine whether cholera outbreaks in Tanzania are from a single or multiple sources, or whether the outbreak strains are genetically related to each other, because these isolates have not yet been characterized by modern genetic methods. Here, we use two established methods, multi-locus variable-number tandem-repeat analysis (MLVA) and whole genome sequencing (WGS), to determine the genetic relatedness and establish transmission patterns of outbreak strains, which can lead to conclusions on the source(s) of these cholera outbreaks. Such conclusions could form the basis for interventions to better control the spread of future outbreaks.

## Methods

### Study setting and study period

**Fig. 1:**
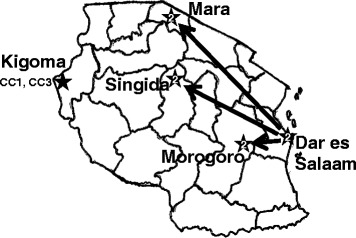
Tanzania map indicating the five geographical regions where *Vibrio cholerae* was isolated between January and November, 2015. The MLVA CC of the isolates at each location is indicated.

Clinical *V. cholerae* isolates were collected from the five regions (Fig. 1) which experienced outbreaks between December 2011 and November 2015. These specimens were collected with consent of the patients, as part of routine surveillance by the Ministry of Health, and were de-identified to protect patient anonymity.

### Isolation of *Vibrio cholerae*

Ninety-six *V. cholerae* isolates preserved in skim milk at −80 °C were revived by inoculation into alkaline peptone water at 37 °C for 6 hours and cultured aerobically on thiosulfate citrate bile salt sucrose medium at 35 °C overnight. One discrete colony from thiosulfate citrate bile salt sucrose medium was subcultured on tryptone soya agar at 35 °C overnight under aerobic conditions to obtain sufficient growth for biochemical and serological tests.

### Biochemical screening and confirmation of the isolates

All isolates were confirmed as positive for oxidase, indole and string tests. The isolates were serogrouped using polyvalent serogroup O1 specific antiserum and confirmed for serotypes using Inaba and Ogawa specific antisera from DENKA SEIKEN Co. Ltd-Japan. Saline controls were included to detect auto agglutination.

### DNA extraction

DNA was extracted from overnight *V. cholerae* growth on Luria Bertani (LB) broth culture medium by taking 5 μL and following the Qiagen-DNeasy DNA extraction kit protocol (Qiagen, Germantown, MD).

### Multiple-locus variable-number tandem-repeat analysis (MLVA)

Five loci (VC0147, VC0436-7, VC1650, VCA0171, and VCA0283) containing a variable number of tandem repeats were amplified using specific primers [1]. These PCR products were separated, detected, and sized by using a 3730xl automatic sequencer using internal lane standards (Liz 600), and the Gene Scan program (all from Applied Biosystems, Foster City, CA, USA). Genotypes were determined using published formulas to calculate the number of repeats from the length of each allele [1]. Five loci were ordered by their chromosomal location and a genotype (e.g., 9, 4, 14, 22, 17) was interpreted as an isolate having alleles of 9, 4, 14, 22, and 17 repeats at the 5 loci, respectively. The relatedness of isolates was assessed by using eBURST version 3 (http://eburst.mlst.net) [1, 4]. Genetically-related genotypes are defined as those possessing identical alleles at 4 of the 5 loci; groups of related genotypes are called MLVA clonal complexes (CCs).

### Genome sequencing

We sequenced the genomes of 39 *V. cholerae* O1 isolates using DNA prepared for Illumina sequencing with the KAPA High Throughput Library Preparation Kit (Kapa Biosystems, Wilmington, MA). DNA was fragmented with the Covaris E210. Libraries were prepared using a modified version of manufacturer’s with-bead protocol (Kapa Biosystems, Wilmington, MA). The libraries were enriched and barcoded by ten cycles of PCR amplification with primers containing an index sequence seven nucleotides in length. The libraries were then sequenced using a 100 bp paired-end run on an Illumina HiSeq2500 (Illumina, San Diego, CA).

### Whole genome alignment and detection of single nucleotide variants (SNVs)

The quality of the 101-base paired-end reads was confirmed using FastQC (http://www.bioinformatics.bbsrc.ac.uk/projects/fastqc). High quality reads were assembled with “Spades” software (v.3.6.2) [5], using the options ‘–careful’ to reduce the number of mis-assemblies and ‘–cov-cutoff auto’ to remove the potentially mis-assembled low coverage contigs. Annotation was performed using the RAST server [6]. The assembled annotated files are in Genbank. Nucleotide variation was identified compared to *V. cholerae* O1 El Tor strain TEM/25/01-004 (whole genome sequence tag TANZ_56) in order to avoid spurious single nucleotides variants (SNVs). PARSNP (v1.2) [7] was used to extract and align the variable nucleotides from the core-genome, using the parameter ‘–c’ to constrain the use of all input genomes and generate the ‘.vcf’ variant description file and ‘.ggr’ alignment description file. The ‘.ggr’ file was loaded in Gingr (v1.2) [7] to visualize the alignments and export the variant nucleotide alignment ‘.mfa’ file. The ‘.vcf’ file was then used to remove all variants from the ‘.mfa’ file detected in the edge of the contigs (less than 1 kb of the contigs edges) using an in-house script. FastTree2 (v2.1.9) [8] was used with the default parameters to generate the maximum-likelihood newick tree file using the corrected ‘.mfa’ alignment file. Then iTOL (http://itol.embl.de/) [9] was used to visualize the maximum-likelihood tree. In order to place the isolates into the 7th pandemic phylogeny, we mapped the reads to the *Vibrio cholerae* 01 El Tor reference N16961 using SMALT (http://www.sanger.ac.uk/resources/software/smalt) [10]. The alignment was then striped of putative recombinant sites via Gubbins [11]. The resulting alignment of 5020 nucleotides was used to infer the phylogenic tree using RAxML [12] under the GTR model with 100 bootstrap replicates. The pre-seventh pandemic strain M66 (NCBI accession numbers CP001233 and CP001234) was used as outgroup. The whole genome sequences from previous publications are listed in Additional file 1: Table S1.

## Results

### Multi-locus variable-number tandem-repeat analysis (MLVA)

Our MLVA revealed extensive genetic diversity among the 96 *V. cholerae* O1 isolates (Additional file 2: Table S2). All loci exhibited substantial variation: VC0147 had four alleles; VC436–7, five alleles; VC1650, three alleles; VCA0171, nine alleles; and VCA0283, thirteen alleles. When each isolate was assigned a genotype (on the basis of and in order of the number of repeat units at each locus), we identified 48 genotypes. Further exploration to determine the genetic relatedness of these genotypes revealed three MLVA-CCs, each comprising genotypes that differed from the other genotypes by a single allelic change. In addition, we detected three singleton genotypes that were unrelated to any other genotype; that is, they differed at >2 of the 5 MLVA loci from all other genotypes.

**Fig. 2:**
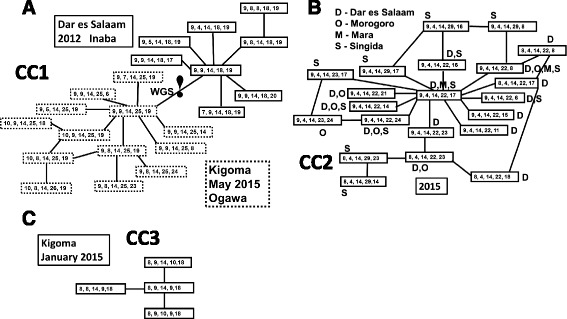
Distribution genotypes in MLVA clonal complexes depicting the genetic relatedness of the *V. cholerae* O1. Genotypes identified in **a**) CC1, two outbreaks: Dar es Salaam in 2011/2012 (boxes with solid lines) and Kigoma in May, 2015 (boxes with dotted lines). The symbol WGS! indicates the location of two genetic characteristics that differentiated between the two portions of the diagram. Those characteristics were serotype: Inaba versus Ogawa and a large (>70 SNVs) difference in the WGS. The relatedness of genotypes is diagrammed in **b**) for CC2, the August – November 2015 outbreak and **c**) for CC3, the January 2015 outbreak in Kigoma

The three genetically separate MLVA-CCs were also geographically and temporally distinct. Clonal complex 1 consisted of 31 isolates having 21 different genotypes (Fig. 2a). Thirteen of the isolates and eight genotypes were from the 2011/2012 outbreak in Dar es Salaam. The other eighteen isolates and thirteen genotypes were from the May, 2015 outbreak in Kigoma refugee camp. In each outbreak, there is a central genotype with many single locus variants and a few of those variants have additional related variants. Of note, despite differing at single MLVA locus, the two outbreaks have distinct genetic characteristics. First, at VCA0171 (locus 4), the 2011–2012 outbreak isolates have allele 18, while the 2015 isolates have allele 25 (with one exception that is 26). The second characteristic is serotypes: the 2011–2012 were Inaba, while the 2015s were Ogawa. These genetic differences also corresponded to a major difference in SNVs (marked by WGS! in Fig. 2a, additional details below). CC2 was comprised of 21 different genotypes distributed among 54 isolates (Fig. 2b). All the isolates were collected from the outbreak that started in Dar es Salaam in August 2015 and spread across multiple regions of Tanzania: Morogoro, later in August; Mara, in September; and Singida, in October. Of note, 47 isolates had allele 22, 4 had allele 23 and the five isolates that had allele 29 were collected in Singida. CC3 was composed of four of the other seven genotypes was found only in January 2015 in Kigoma (Fig. 2c). All but one of the isolates had allele 9 at VCA0171 and the exception had allele 10. The other three genotypes were singletons unrelated to each other or any MLVA-CC. One singleton genotype was collected in January 2015 in Kigoma. The other two singletons were collected in January 2012 in Dar es Salaam.

### Whole genome sequences

We analysed the phylogenetic relationships of the whole genome sequences from 39 *V. cholerae* O1 isolates. The average number of assembled contigs or scaffolds was 136 with an average depth of 386 reads (range 271 to 646). The genomes averaged 4.03 Mb in length and had an average GC content of 47.5%. In order to examine only the highest quality SNVs, we analysed only SNVs that were more than 1 kb from the ends of a contig. The resulting number of SNVs was 196 (Additional file 3: Table S3).

**Fig. 3:**
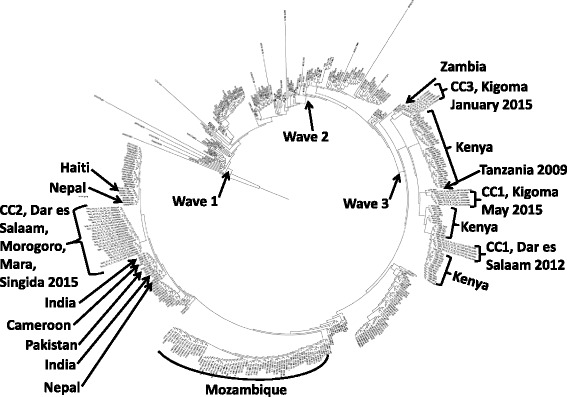
The 7^th^ pandemic *V. cholerae* phylogenetic tree depicting the genetic relatedness and position of Tanzanian isolates among selected other sequenced *V. cholerae* isolates (Additional file 1: Table S1). The phylogenetic tree was constructed on an alignment of the variable sites of 445 strains with predicted recombination sites removed consistent with previous publications [15]. The location and date of collection of selected isolates is noted

**Fig. 4:**
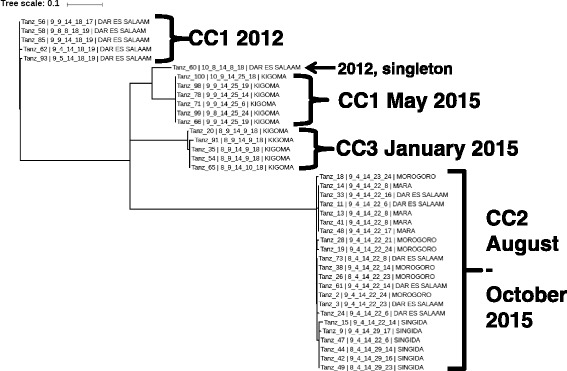
Maximum likelihood tree of genotypes from 39 Tanzanian isolates. Each isolate is identified by a number, its MLVA genotype and the location where it was collected. The date of collection and MLVA CC are assigned to the four clades

Whole genome phylogeny unequivocally confirmed that the current Tanzanian isolates are members of wave 3 within the ongoing seventh pandemic (Fig. 3). Isolates from CC1 & 3 cluster with isolates from Kenya and Zambia. Of note, the Dar es Salaam – Kigoma clade within CC1 groups with a previously sequenced isolate (4784) from Tanzania from 2009. Isolates from CC2 are most closely related to isolates from South Asia and Haiti. None of the Tanzanian isolates are related to the isolates from the neighbouring country, Mozambique. The SNVs discriminated between the isolates in this study in a phylogenetically informative manner. The pairwise differences between WGS genotypes ranged from 0 to 124 SNVs. There were 20 SNVs that varied in only one isolate, nine of the nucleotides occurred in one isolate (Tanz_60) and 7 nucleotides occurred in 6 isolates from the August 2015 outbreak. Maximum likelihood analysis of the WGS data revealed four distinct clades (Fig. 4). Each clade is separated by at least 52 polymorphic nucleotides. The four clades contained isolates from: 1) CC1 in Dar es Salaam in 2012, 2) CC1 in Kigoma during May 2015, 3) CC3 in Kigoma in January 2015 and 4) CC2 in August to October 2015. The two CC1 clusters were separated at least 73 SNVs consistent with their separation by serotype, Inaba or Ogawa and their allelic difference at VCA0171, the fourth locus. The maximum distance within each of the four clades was 2, 0, 3 and 4 SNVs respectively. Closer inspection of the CC2 clade revealed that all the genotypes from Mara did not differ and were identical to two genotypes from Dar es Salaam; three genotypes from Morogoro were identical to themselves and to three genotypes from Dar es Salaam and differed from the other three Morogoro genotypes by a one SNV; four of the Singida genotypes were identical and differed all the other genotypes from every location by one or two nucleotides.

## Discussion

Cholera outbreaks in Tanzania were caused by genetically diverse isolates of *V. cholerae* O1, although within an outbreak there was very little genetic diversity. Among our set of 96 isolates, MLVA revealed 48 genotypes of which 45 were grouped into three MLVA-CCs of genetically related isolates. Among the 39 isolates analysed by WGS, 196 SNVs were observed. Our maximum likelihood analyses of the genome sequences of the 39 isolates revealed four clades each within the 3^rd^ wave of the 7^th^ pandemic [13]. The isolates in each clade, as defined by WGS corresponded to isolates from one of the MLVA-CCs. CC1 was divided into two clades reflecting the distinct outbreaks in different locations, different years and with different serotypes. The isolates within each outbreak primarily belonged to a single MLVA CC and the WGS clade.

Our MLVA data provided two unusual observations. First, in our data, the allele at locus VCA0171 was strongly correlated with the outbreak. In contrast, most previous MLVA investigations have found that the two small chromosome loci VCA0171 and VCA0283 were highly variable and did not correlate with any variable [1, 14]. Second, our CC1 was not a single clade when analysed by WGS. This contrasts with previous observations that i) isolates from the same CC many miles apart were part of the same clade by WGS [14] or ii) closely related WGS sequences may have unrelated MLVA genotypes [14, 15]. Whether this is the result of convergence or a slower than expected divergence of the MLVA genotypes cannot be determined from our data.

The epidemiological and genetic data, MLVA and WGS, are all consistent with three separate outbreaks in Tanzania during 2015. The first in January 2015 occurred in Kigoma and was caused by isolates from CC3 and a distinct singleton genotype representative of another genetic lineage of *V. cholerae*. The second outbreak in May also in Kigoma was caused by isolates from CC1. This indicates that the two outbreaks in Kigoma, occurring 4 months apart, were each caused by independent genetic lineages. Consistent with a single source, the isolates within each outbreak were less than 5 SNVs apart. The genomes from MLVA CC1 & 3 are likely the ongoing spread of the lineage (or lineages) that was imported into Africa between 1993 and 2002 [16]. The third, August 2015 outbreak includes only isolates from MLVA CC2, again a single source for the isolates is consistent with them being fewer than five nucleotides apart. This outbreak has spread from Dar es Salaam in August to Morogoro later August, to Mara and then Singida (see Fig. 1). Genotypes from the two of the three outbreaks have been observed in or related to isolates from Dar es Salaam, we hypothesize that Dar es Salaam may be a reservoir of *Vibrio cholerae* in Tanzania*.*

## Conclusion

Each cholera outbreak in Tanzania in 2015 was caused by a genetic distinct group of closely related isolates. Each outbreak has a single primary source and interventions should focus on stopping the spread of the disease. These outbreaks are part of the ongoing spread of cholera through Africa, as clearly seen with these strains related to the Zambian, Kenyan and other African lineages. Dar es Salaam is hypothesized to be the hub of cholera outbreaks in Tanzania, controlling cholera in Dar es Salaam will have a major impact on cholera outbreaks in the country.

## Additional files

Additional file 1: Isolates for 7th Pandemic Tree. (XLSX 20 kb)
Additional file 2: Multiple alignment file of 196 SNVs. (DOCX 13 kb)
Additional file 3: Metadata, MLVA genotypes and whole genome sequence charateristics for isolates from Tanzania analyzed in this manuscript. (DOCX 19 kb)

## Abbreviations

CC: Clonal complex
DNA: deoxyribonucleic acid
MLVA: Multilocus variable tandem repeat analysis
SNV: single nucleotide variants
*V.*: *Vibrio*
WGS: Whole genome sequencing
