# Supplementary material for: Genetic Characterization of Vibrio cholerae O1 isolates from outbreaks between 2011 and 2015 in Tanzania
Source: BMC Infect Dis. 2017 Feb 20;17:157. doi: 10.1186/s12879-017-2252-9 (PMC5319185; doi:10.1186/s12879-017-2252-9)
Supplement: Additional file 2: — Multiple alignment file of 196 SNVs. (DOCX 13 kb) [file 12879_2017_2252_MOESM2_ESM.docx]

Supplemental Table 2: Multiple alignment file of 196 SNVs

>Tanz_91

CGGCGGCCGAGACACTCGACCTTGAGAGAAGTCACCCCCTATCCGGTCGAGCCTGCGACAGGGACACTTCAAAAGGTGTTGTGATACCCTGACCGGCAGTATGCCGGCTGCAGTCACACGATTAGAAGACTGAACACTCAGGCGGGCTGGCGGCTCTCCAAGGTCAGAGCGAGCTTCTTCTAATGGGCCTAGACCA

>Tanz_100_1

CGGAAGCCGGGGAACTTAACCTTGAAAAAGATAGTCTCCCGTCCGGTCGGGCCTGCGATAGGGACGCGACGACAGGTATCGTGATGCCCTGGCCAGTAGTATGCCGGCTGCAGGCACACGATCAAGCGACTGACTACTCCTGCGGGTTGGCTATTCCCCAAGGTTAGAACAGGTCTCTTCGACCACGCCTAGACCA

>Tanz_98

CGGAAGCCGGGGAACTTAACCTTGAAAAAGATAGTCTCCCGTCCGGTCGGGCCTGCGATAGGGACGCGACGACAGGTATCGTGATGCCCTGGCCAGTAGTATGCCGGCTGCAGGCACACGATCAAGCGACTGACTACTCCTGCGGGTTGGCTATTCCCCAAGGTTAGAACAGGTCTCTTCGACCACGCCTAGACCA

>Tanz_14

CGAAGGTAGAGGCATTCGACCATAAGAGGAGCCGCCCTCCGTCCTACTGGGTTTGTACTGAGGACGTGAAGGCGGTCGCCACACTGCCTGGGCTGGCCGCACATCAGTTGTATGGACACGAACCAGCGGAGAGCCATTAAGGTGGTCCGATTGTCTCAAAAGGCTGGCGTAAACCACCCTGACTACGCCCGAACCA

>Tanz_62

AGGAGACCAAAGCACACGCTTATGTGAGAAGTCGCCCCTCGCACGGTCAGACCGACGATAGGAACGCGACGACAAGTGTCGTGAAGTTCTGGTCGGCAATATGCAGACGGCGGGCAACCACTCAAGCAACTGACCGCCCAGACGAGCTTGCTGTTCCCCGTAATTAGAGCAAGCCTCTTCGGCTACATTTAGGAAG

>Tanz_73

CGAAGGTAGAGGCATTCGACCATAAGAGGAGCCGCCCTCCGTCCTACTGGGTTTGTACTGAGGACGTGAAGGCGGTCGCCACACTGCCTGGGCTGGCCGCACATCAGTTGTATGGACACGATCCAGCGGAGAGCCATTAAGGTGGTCCGATTGTCTCAAAAGGCTGGCGTAAACCACCCTGACTACGCCCGAACCA

>Tanz_33

CGAAGGTAGAGGCATTCGACCATAAGAGGAGCCGCCCTCCGTCCTACTGGGTTTGTACTGAGGACGTGAAGGCGGTCGCCACACTGCCTGGGCTGGCCGCACATCAGTTGTATGGACACGAACCAGCGGAGAGCCATTAAGGTGGTCCGATTGTCTCAAAAGGCTGGCGTAAACCACCCTGACTACGCCCGAACCA

>Tanz_93

AGGAGACCAAAGCACACGCTTATGTGAGAAGTCGCCCCTCGCACGGTCAGACCGACGATAGGAACGCGACGACAAGTGTCGTGAAGTTCTGGTCGGCAATATGCAGACGGCGGGCAAACACTCAAGCAACTGACCGCCCAGACAAGCTTGCTGTTCCCCGTAATTAGAGCAAGCCTCTTCGGCTACATTTAGGAAG

>Tanz_35

CGGCGGCCGAGACACTCGACCTTGAGAGAAGTCACCCCCTATCCGGTCGAGCCTGCGACAGGGACACGACAAAAGGTGTTGTGATACCCTGACCGGCAGTATGCCGGCTGCAGTCACACGATTAGAAGACTGAACACTCAGGCGGGCTGGCGGCTCTCCAAGGTCAGAGCGAGCTTCTTCTAATGGGCCTAGACCA

>Tanz_38

CGAAGGTAGAGGCATTCGACCATAAGAGGAGCCGCCCTCCGTCCTACTGGGTTTGTACTGAGGACGTGAAGGCGGTCGCCACACTGCCTGGGCTGGCCGCACATCAGTTGTATGGACACGATCCAGCGGAGAGCCATTAAGGTGGTCCGATTGTCTCAAAAGGCTGGCGTAAACCACCCTGACTACGCCCGAACCA

>Tanz_54

CGGCGGCCGAGACACTCGACCTTGAGAGAAGTCACCCCCTATCCGGTCGAGCCTGCGACAGGGACACGACAAAAGGTGTTGTGATACCCTGACCGGCAGTATGCCGGCTGCAGTCACACGATTAGAAGACTGAACACTCAGGCGGGCTGGCGGCTCTCCAAGGTCAGAGCGAGCTTCTTCTAATGGGCCTAGACCA

>Tanz_18

CGAAGGTAGAGGCATTCGACCATAAGAGGAGCCGCCCTCCGTCCTACTGGGTTTGTACTGAGGGCGTGAAGGCGGTCGCCACACTGCCTGGGCTGGCCGCACATCAGTTGTATGGACACGATCCAGCGGAGAGCCATTAAGGTGGTCCGATTGTCTCAAAAGGCTGGCGTAAACCACCCTGACTACGCCCGAACCA

>Tanz_19

CGAAGGTAGAGGCATTCGACCATAAGAGGAGCCGCCCTCCGTCCTACTGGGTTTGTACTGAGGACGTGAAGGCGGTCGCCACACTGCCTGGGCTGGCCGCGCATCAGTTGTATGGACACGATCCAGCGGAGAGCCATTAAGGTGGTCCGATTGTCTCAAAAGGCTGGCGTAAACCACCCTGACTACGCCCGAACCA

>Tanz_56

AGGAGACCAAAGCACACGCTTATGTGAGAAGTCGCCCCTCGCACGGTCAGACCGACGATAGGAACGCGACGACAAGTGTCGTGAAGTTCTGGTCGGCAATATGCAGACGGCGGGCAAACACTCAAGCAACTGACCGCCCAGACGAGCTTGCTGTTCCCCGTAATTAGAGCAAGCCTCTTCGGCTACATTTAGGAAG

>Tanz_78

CGGAAGCCGGGGAACTTAACCTTGAAAAAGATAGTCTCCCGTCCGGTCGGGCCTGCGATAGGGACGCGACGACAGGTATCGTGATGCCCTGGCCAGTAGTATGCCGGCTGCAGGCACACGATCAAGCGACTGACTACTCCTGCGGGTTGGCTATTCCCCAAGGTTAGAACAGGTCTCTTCGACCACGCCTAGACCA

>Tanz_11

CGAAGGTAGAGGCATTCGACCATAAGAGGAGCCGCCCTCCGTCCTACTGGGTTTGTACTGAGGACGTGAAGGCGGTCGCCACACTGCCTGGGCTGGCCGCACATCAGTTGTATGGACACGAACCAGCGGAGAGCCATTAAGGTGGTCCGATTGTCTCAAAAGGCTGGCGTAAACCACCCTGACTACGCCCGAACCA

>Tanz_13

CGAAGGTAGAGGCATTCGACCATAAGAGGAGCCGCCCTCCGTCCTACTGGGTTTGTACTGAGGACGTGAAGGCGGTCGCCACACTGCCTGGGCTGGCCGCACATCAGTTGTATGGACACGAACCAGCGGAGAGCCATTAAGGTGGTCCGATTGTCTCAAAAGGCTGGCGTAAACCACCCTGACTACGCCCGAACCA

>Tanz_58

AGGAGACCAAAGCACACGCTTATGTGAGAAGTCGCCCCTCGCACGGTCAGACCGACGATAGGAACGCGACGACAAGTGTCGTGAAGTTCTGGTCGGCAATATGCAGACGGCGGGCAAACACTCAAGCAACTGACCGCCCAGACGAGCTTGCTGTTCCCCGTAATTAGAGCAAGCCTCTTCGGCTACATTTAGGAAG

>Tanz_15

CGAAGGTAGAGGCATTCGACCATAAGAGGAGCCGCACTCCGTCCTACTGGGTTTGTACTGAAGACGTGAAGGCGGTCGCCACACTGCCTGGGCTGGCCGCACATCAGTTGTATGGTCACGATCCAGCGGAGAGCCATTAAGGTGGTCCGATTGTCTCAAAAGGCTGGCGTAAACCACCCTGACTACGCCCGAACCA

>Tanz_71

CGGAAGCCGGGGAACTTAACCTTGAAAAAGATAGTCTCCCGTCCGGTCGGGCCTGCGATAGGGACGCGACGACAGGTATCGTGATGCCCTGGCCAGTAGTATGCCGGCTGCAGGCACACGATCAAGCGACTGACTACTCCTGCGGGTTGGCTATTCCCCAAGGTTAGAACAGGTCTCTTCGACCACGCCTAGACCA

>Tanz_99

CGGAAGCCGGGGAACTTAACCTTGAAAAAGATAGTCTCCCGTCCGGTCGGGCCTGCGATAGGGACGCGACGACAGGTATCGTGATGCCCTGGCCAGTAGTATGCCGGCTGCAGGCACACGATCAAGCGACTGACTACTCCTGCGGGTTGGCTATTCCCCAAGGTTAGAACAGGTCTCTTCGACCACGCCTAGACCA

>Tanz_47

CGAAGGTAGAGGCATTCGACCATAAGAGGAGCCGCCCTCCGTCCTACTGGGTTTGTACTGAAGACGTGAAGGCGGTCGCCACACTGCCTGGGCTGGCCGCACATCAGTTGTATGGACACGATCCAGCGGAGAGCCATTAAGGTGGTCCGATTGTCTCAAAAGGCTGGCGTAAACCACCCTGACTACGCCCGAACCA

>Tanz_85

AGGAGACCAAAGCACACGCTTATGTGAGAAGTCGCCCCTCGCACGGTCAGACCGACGATAGGAACGCGACGACAAGTGTCGTGAAGTTCTGGTCGGCAATATGCAGACGGCGGGCAAACACTCAAGCAACTGACCGCCCAGACGAGCTTGCTGTTCCCCGTAATTAGAGCAAGCCTCTTCGGCTACATTTAGGAAG

>Tanz_24

CGAAGGTAGAGGCGTTCGACCATAAGAGGAGCCGCCCTCCGTCCTACTGGGTTTGTACTGAGGACGTGAAGGCGGTCGCCACACTGCCTGGGCTGGCCGCACATCAGTTGTATGGACACGATCCAGCGGAGAGCCATTAAGGTGGTCCGATTGTCTCAAAAGGCTGGCGTAAACCACCCTGACTACGCCCGAACCA

>Tanz_26

CGAAGGTAGAGGCATTCGACCATAAGAGGAGCCGCCCTCCGTCCTACTGGGTTTGTACTGAGGACGTGAAGGCGGTCGCCACACTGCCTGGGCTGGCCGCACATCAGTTGTATGGACACGATCCAGCGGAGAGCCATTAAGGTGGTCCGATTGTCTCAAAAGGCTGGCGTAAACCACCCTGACTACGCCCGAACCA

>Tanz_20

CGGCGGCCGAGACACTCGACCTTGAGAGAAGTCACCCCCTATCCGGTCGAGCCTGCGACAGGGACACGACAAAAGGTGTTGTGATACCCTGACCGGCAGTATGCCGGCTGCAGTCACACGATTAGAAGACTGAACACTCAGGCGGGCTGGCGGTTCTCCAAGGTCAGAGCGAGCTTCTTCTAATGGGCCTAGACCA

>Tanz_28

CGAAGGTAGAGGCATTCGACCAAAAGAGGAGCCGCCCTCCGTCCTACTGGGTTTGTACTGAGGACGTGAAGGCGGTCGCCACACTGCCTGGGCTGGCCGCACATCAGTTGTATGGACACGATCCAGCGGAGAGCCATTAAGGTGGTCCGATTGTCTCAAAAGGCTGGCGTAAACCACCCTGACTACGCCCGAACCA

>Tanz_61

CGAAGGTAGAGGCATTCGACCATAAGAGGAGCCGCCCTCCGTCCTACTGGGTTTGTACTGAGGACGTGAAGGCGGTCGCCACACTGCCTGGGCTGGCCGCACATCAGTTGTATGGACACGATCCAGCGGAGAGCCATTAAGGTGGTCCGATTGTCTCAAAAGGCTGGCGTAAACCACCCTGACTACGCCCGAACCA

>Tanz_60

CAGAGGCCGAGGAACTTAACCTTGAAAAAGGTAGCCCCCCGTCTGGTCGGGCCTGCGATAGGGATGCGACGACAGGTATCGTGATGCCCTAGCCAACAGTATGCCGGCTACGGGCACATGATCAAGCGACTGACCACTCAGGCGGGTTGGCTGTTCCCCAAGGTTAAAACAAGCCTTTTCGACCACGCCTAGACCA

>Tanz_44

CGAAGGTAGAGGCATTCGACCATAAGAGGAGCCGCCCTCCGTCCTACTGGGTTTGTACTGAAGACGTGAAGGCGGTCGCCACACTGCCTGGGCTGGCCGCACATCAGTTGTATGGACACGATCCAGCGGAGAGCCATTAAGGTGGTCCGATTGTCTCAAAAGGCTGGCGTAAACCACCCTGACTACGCCCGAACCA

>Tanz_65

CGGCGGCCGAGACACTCGACCTTGAGAGAAGTCACCCCCTATCCGGTCGAGCCTGCGACAGGGACACGACAAAAGGTGTTGTGATACCCTGACCGGCAGTATGCCGGCTGCAGTCACACGATTAGAAGACTGAACACTCAGGCGGGCTGGCGGCTCTCCAAGGTCAGAGCGAGCTTCTTCTAATGGGCCTAGACCA

>Tanz_42

CGAAGGTAGAGGCATTCGACCATAAGAGGAGCCGCCCTCCGTCCTACTGGGTTTGTACTGAAGACGTGAAGGCGGTCGCCACACTGCCTGGGCTGGCCGCACATCAGTTGTATGGACACGATCCAGCGGAGAGCCATTAAGGTGGTCCGATTGTCTCAAAAGGCTGGCGTAAACCACCCTGACTACGCCCGAACCA

>Tanz_41

CGAAGGTAGAGGCATTCGACCATAAGAGGAGCCGCCCTCCGTCCTACTGGGTTTGTACTGAGGACGTGAAGGCGGTCGCCACACTGCCTGGGCTGGCCGCACATCAGTTGTATGGACACGAACCAGCGGAGAGCCATTAAGGTGGTCCGATTGTCTCAAAAGGCTGGCGTAAACCACCCTGACTACGCCCGAACCA

>Tanz_66

CGGAAGCCGGGGAACTTAACCTTGAAAAAGATAGTCTCCCGTCCGGTCGGGCCTGCGATAGGGACGCGACGACAGGTATCGTGATGCCCTGGCCAGTAGTATGCCGGCTGCAGGCACACGATCAAGCGACTGACTACTCCTGCGGGTTGGCTATTCCCCAAGGTTAGAACAGGTCTCTTCGACCACGCCTAGACCA

>Tanz_49

CGAAGGTAGAGGCATTCGACCATAAGAGGAGCCGCCCTCCGTCCTACTGGGTTTGTACTGAAGACGTGAAGGCGGTCGCCACACTGCCTGGGCTGGCCGCACATCAGTTGTATGGACACGATCCAGCGGAGAGCCATTAAGGTGGTCCGATTGTCTCAAAAGGCTGGCGTAAACCACCCTGACTACGCCCGAACCA

>Tanz_48

CGAAGGTAGAGGCATTCGACCATAAGAGGAGCCGCCCTCCGTCCTACTGGGTTTGTACTGAGGACGTGAAGGCGGTCGCCACACTGCCTGGGCTGGCCGCACATCAGTTGTATGGACACGAACCAGCGGAGAGCCATTAAGGTGGTCCGATTGTCTCAAAAGGCTGGCGTAAACCACCCTGACTACGCCCGAACCA

>Tanz_2

CGAAGGTAGAGGCATTCGACCATAAGAGGAGCCGCCCTCCGTCCTACTGGGTTTGTACTGAGGACGTGAAGGCGGTCGCCACACTGCCTGGGCTGGCCGCACATCAGTTGTATGGACACGATCCAGCGGAGAGCCATTAAGGTGGTCCGATTGTCTCAAAAGGCTGGCGTAAACCACCCTGACTACGCCCGAACCA

>Tanz_3

CGAAGGTAGAGGCATTCGACCATAAGAGGAGCCGCCCTCCGTCCTACTGGGTTTGTACTGAGGACGTGAAGGCGGTCGCCACACTGCCTGGGCTGGCCGCACATCAGTTGTATGGACACGATCCAGCGGAGAGCCATTAAGGTGGTCCGATTGTCTCAAAAGGCTGGCGTAAACCACCCTGACTACGCCCGAACCA

>Tanz_9

CGAAGGTAGAGGCATTCGACCATAAGGGGAGCCGCCCTCCGTCCTACTGGGTTTGTACTGAAGACGTGAAGGCGGTCGCCACACTGCCTGGGCTGGCCGCACATCAGTTGTATGGACACGATCCAGCGGAGAGCCATTAAGGTGGTCCGATTGTCTCAAAAGGCTGGCGTAAACCACCCTGACTACGCCCGAACCA
