# Supplementary material for: Genetic Characterization of Vibrio cholerae O1 isolates from outbreaks between 2011 and 2015 in Tanzania
Source: BMC Infect Dis. 2017 Feb 20;17:157. doi: 10.1186/s12879-017-2252-9 (PMC5319185; doi:10.1186/s12879-017-2252-9)
Supplement: Additional file 3: — Metadata, MLVA genotypes and whole genome sequence charateristics for isolates from Tanzania analyzed in this manuscript. (DOCX 19 kb) [file 12879_2017_2252_MOESM3_ESM.docx]

ISOLATE ID Region Month Year MLVA CC VC0147 VC0436 VC1650 VCA0171 VCA0283 Genome ID Accession Number Average scaffold coverage "Scaffold

Number" "Number

Complete

Genes"

48055 DAR ES^1^ AUGUST 2015 2 9 4 14 22 14 Tanz_61 SAMN06144963 646.15 117 3568

1 MOROGORO AUGUST 2015 2 9 4 14 22 24 Tanz_2 SAMN06144937 339.91 119 3554

39361 DAR ES^1^ AUGUST 2015 2 8 4 14 22 8 Tanz_73 SAMN06144968 377.06 120 3565

11S SINGIDA OCTOBER 2015 2 8 4 14 29 14 Tanz_44 SAMN06144955 347.98 123 3561

43 KIGOMA JANUARY 2015 3 8 9 14 9 18 Tanz_91 SAMN06144971 374.39 123 3587

20478 KIGOMA MAY 2015 1 9 9 14 25 19 Tanz_66 SAMN06144966 420.19 124 3585

21027 KIGOMA MAY 2015 1 9 9 14 25 6 Tanz_71 SAMN06144967 468.65 126 3582

OO4 DAR ES^1^ AUGUST 2015 2 9 4 14 22 23 Tanz_3 SAMN06144938 311.68 126 3562

7 MOROGORO AUGUST 2015 2 9 4 14 23 24 Tanz_18 SAMN06144944 271.93 127 3561

O5S SINGIDA OCTOBER 2015 2 9 4 14 29 17 Tanz_9 SAMN06144939 339.68 127 3559

21B KIGOMA JANUARY 2015 3 8 9 14 10 18 Tanz_65 SAMN06144965 448.98 127 3588

O9S SINGIDA OCTOBER 2015 2 8 4 14 29 23 Tanz_49 SAMN06144958 377.71 128 3559

5 MOROGORO AUGUST 2015 2 8 4 14 22 23 Tanz_26 SAMN06144948 352.1 129 3559

9 MOROGORO AUGUST 2015 2 9 4 14 22 24 Tanz_19 SAMN06144945 369.37 130 3559

O7S SINGIDA OCTOBER 2015 2 9 4 14 29 16 Tanz_42 SAMN06144954 307.22 131 3560

O3S SINGIDA OCTOBER 2015 2 9 4 14 22 14 Tanz_15 SAMN06144943 343.7 132 3557

20390 KIGOMA MAY 2015 1 9 9 14 25 14 Tanz_78 SAMN06144969 390.01 133 3584

O5MU MARA SEPTEMBER 2015 2 9 4 14 22 8 Tanz_13 SAMN06144941 280.29 133 3558

2 MOROGORO AUGUST 2015 2 9 4 14 22 14 Tanz_38 SAMN06144952 365.48 134 3560

O2 KIGOMA JANUARY 2015 3 8 9 14 9 18 Tanz_54 SAMN06144959 369.14 136 3578

O1S SINGIDA OCTOBER 2015 2 9 4 14 22 6 Tanz_47 SAMN06144956 294.54 158 3560

8 MOROGORO AUGUST 2015 2 9 4 14 22 21 Tanz_28 SAMN06144949 370.11 159 3558

7714 DAR ES^1^ AUGUST 2015 2 9 4 14 22 6 Tanz_11 SAMN06144940 380.29 202 3568

39 KIGOMA JANUARY 2015 3 8 9 14 9 18 Tanz_20 SAMN06144946 76.17 134 3571

O3MU MARA SEPTEMBER 2015 2 9 4 14 22 8 Tanz_14 SAMN06144942 163.06 125 3556

O7MU MARA SEPTEMBER 2015 2 9 4 14 22 8 Tanz_41 SAMN06144953 83.4 109 3560

47610 DAR ES^1^ AUGUST 2015 2 9 4 14 22 6 Tanz_24 SAMN06144947 143.98 118 3553

J8YRS KIGOMA MAY 2015 1 9 9 14 25 19 Tanz_98 SAMN06144973 137.76 115 3576

19886 KIGOMA MAY 2015 1 9 8 14 25 24 Tanz_99 SAMN06144974 119.67 111 3567

17609 KIGOMA MAY 2015 1 10 9 14 25 18 Tanz_100 SAMN06144975 183.71 115 3582

K-G2-6 KIGOMA MAY 2015 1 9 9 14 25 19

21059 KIGOMA MAY 2015 1 9 9 14 25 8

20698 KIGOMA MAY 2015 1 9 9 14 25 19

KG302 KIGOMA MAY 2015 1 9 5 14 25 19

20464 KIGOMA MAY 2015 1 9 5 14 25 19

20546 KIGOMA MAY 2015 1 9 8 14 25 19

20547 KIGOMA MAY 2015 1 10 8 14 26 19

21055 KIGOMA MAY 2015 1 10 8 14 25 19

21058 KIGOMA MAY 2015 1 9 9 14 25 6

20849 KIGOMA MAY 2015 1 9 8 14 25 23

KG303 KIGOMA MAY 2015 1 10 9 14 25 19

19954 KIGOMA MAY 2015 1 9 7 14 25 19

21054 KIGOMA MAY 2015 1 10 9 14 25 19

7649 DAR ES^1^ AUGUST 2015 2 9 4 14 22 6

48087 DAR ES^1^ AUGUST 2015 2 9 4 14 22 6

48070 DAR ES^1^ AUGUST 2015 2 9 4 14 22 6

80 DAR ES^1^ AUGUST 2015 2 9 4 14 22 8

38487 DAR ES^1^ AUGUST 2015 2 9 4 14 22 8

47633 DAR ES^1^ AUGUST 2015 2 9 4 14 22 11

100 DAR ES^1^ AUGUST 2015 2 9 4 14 22 14

48068 DAR ES^1^ AUGUST 2015 2 9 4 14 22 14

39497 DAR ES^1^ AUGUST 2015 2 9 4 14 22 14

48015 DAR ES^1^ AUGUST 2015 2 9 4 14 22 15

39357 DAR ES^1^ AUGUST 2015 2 9 4 14 22 15

47984 DAR ES^1^ AUGUST 2015 2 9 4 14 22 16

47595 DAR ES^1^ AUGUST 2015 2 8 4 14 22 17

47967 DAR ES^1^ AUGUST 2015 2 9 4 14 22 17

47994 DAR ES^1^ AUGUST 2015 2 9 4 14 22 17

47930 DAR ES^1^ AUGUST 2015 2 8 4 14 22 18

48051 DAR ES^1^ AUGUST 2015 2 9 4 14 22 21

47592 DAR ES^1^ AUGUST 2015 2 8 4 14 22 23

38484 DAR ES^1^ AUGUST 2015 2 9 4 14 22 23

47577 DAR ES^1^ AUGUST 2015 2 9 4 14 22 23

47962 DAR ES^1^ AUGUST 2015 2 9 4 14 22 23

5 DAR ES^1^ AUGUST 2015 2 9 4 14 22 23

39360 DAR ES^1^ AUGUST 2015 2 9 4 14 22 23

200-1 DAR ES^1^ AUGUST 2015 2 9 4 14 22 23

7720 DAR ES^1^ AUGUST 2015 2 9 4 14 22 24

10 MOROGORO AUGUST 2015 2 9 4 14 22 8

O2S SINGIDA OCTOBER 2015 2 9 4 14 29 8

O8S SINGIDA OCTOBER 2015 2 9 4 14 29 8

O6S SINGIDA OCTOBER 2015 2 9 4 14 22 8

10S SINGIDA OCTOBER 2015 2 9 4 14 22 16

12S SINGIDA OCTOBER 2015 2 9 4 14 23 17

O4S SINGIDA OCTOBER 2015 2 9 4 14 22 24

36 KIGOMA JANUARY 2015 3 8 9 10 9 18

41 KIGOMA JANUARY 2015 3 8 8 14 9 18

TEM/24/01-001 DAR ES^1^ JANUARY 2012 1 9 5 14 18 19

TEM/10/01-004 DAR ES^1^ JANUARY 2012 1 9 8 14 18 19

TEM/15/01-016 DAR ES^1^ JANUARY 2012 1 9 8 14 18 19

TEM/15/01-011 DAR ES^1^ JANUARY 2012 1 7 9 14 18 19

TEM/18/01-003 DAR ES^1^ JANUARY 2012 1 9 8 14 18 19

TEM/15/01-009 DAR ES^1^ JANUARY 2012 1 9 9 14 18 20

TEM/18/01-004 DAR ES^1^ JANUARY 2012 1 9 9 14 18 17

TEM/15/01-012 DAR ES^1^ JANUARY 2012 1 9 4 14 18 19

TEM/10/01-003 DAR ES^1^ JANUARY 2012 S 10 9 14 18 15

38 KIGOMA JANUARY 2015 S 10 9 14 9 6

TEM/25/01-004 DAR ES^1^ JANUARY 2012 1 9 9 14 18 17 Tanz_56 4960^2^ 472.7 139 3581

TEM/12/12-001 DAR ES^1^ DECEMBER 2011 1 9 8 8 18 19 Tanz_58 4961^2^ 410.9 140 3583

47623 DAR ES^1^ AUGUST 2015 2 9 4 14 22 16 Tanz_33 4950^2^ 314.4 145 3558

31 KIGOMA JANUARY 2015 3 8 9 14 9 18 Tanz_35 4951^2^ 317.1 146 3578

TEM/29/01-003 DAR ES^1^ JANUARY 2012 1 9 9 14 18 19 Tanz_85 4970^2^ 435.9 148 3582

TEM/15/01-005 DAR ES^1^ JANUARY 2012 1 9 5 14 18 19 Tanz_93 4972^2^ 485.4 151 3587

O6MU MARA SEPTEMBER 2015 2 9 4 14 22 17 Tanz_48 4957^2^ 374.4 152 3563

TEM/04/01-001 DAR ES^1^ JANUARY 2012 1 9 4 14 18 19 Tanz_62 4964^2^ 466.3 156 3579

TEM/10/01-002 DAR ES^1^ JANUARY 2012 S 10 8 14 8 18 Tanz_60 4962^2^ 537.0 123 3579

Footnotes: ^1^- Dar es Salaam ^2^- SAMN0614should be attached as a prefix to obtain the Genbank number
